# The Comparative Efficacy of Multiple Interventions for Mild Cognitive Impairment in Alzheimer's Disease: A Bayesian Network Meta-Analysis

**DOI:** 10.3389/fnagi.2020.00121

**Published:** 2020-06-05

**Authors:** Xin Lai, Hao Wen, Yu Li, Liming Lu, Chunzhi Tang

**Affiliations:** Medical College of Acu-Moxi and Rehabilitation, Guangzhou University of Chinese Medicine, Guangzhou, China

**Keywords:** acupuncture, Alzheimer's disease, mild cognitive impairment, multiple interventions, music therapy, network meta-analysis

## Abstract

**Background:** Mild cognitive impairment (MCI) is the early phase of Alzheimer's disease (AD). The aim of early intervention for MCI is to decrease the rate of conversion from MCI to AD. However, the efficacy of multiple interventions in MCI, and the optimal methods of delivery, remain controversial. We aimed to compare and rank the treatment methods for MCI in AD, in order to find an optimal intervention for MCI and a way to prevent or delay the occurrence of AD.

**Methods:** Pair-wise and network meta-analysis were conducted to integrate the treatment effectiveness through direct and indirect evidence. Four English databases and three Chinese databases were searched for international registers of eligible published, single or double blind, randomized controlled trials up to September 31st 2019. We included nine comparative interventions: pharmacological therapies which incorporated cholinesterase inhibitors (ChEI), ginkgo, nimodipine, and Chinese medicine; non-pharmacological therapies comprising of acupuncture, music therapy, exercise therapy, and nutrition therapy; and a placebo group. The primary outcome was the Mini-Mental State Examination (MMSE) score. The secondary outcome was the AD Assessment Scale-cognitive subscale (ADAS-cog).

**Results:** Twenty-eight trials were eligible, including 6,863 participants. In the direct meta-analysis, as for the Mini-Mental State Examination scale, the ChEIs (MD: −0.38; 95% CI: −0.74, −0.01), Chinese medicine (MD: −0.31; 95% CI: −0.75, 0.13), exercise therapy (MD: −0.50; 95% CI: −0.65, −0.35), music therapy (MD: −1.71; 95% CI: −4.49, 1.07), were statistically more efficient than placebo. For AD Assessment Scalecognitive subscale outcome, ChEIs (MD: 1.20; 95% CI: 0.73, 1.68), Acupuncture (MD: 1.36; 95% CI: 1.28, 1.44), Chinese medicine (MD: 0.61; 95% CI: 0.49, 0.73) and exercise (MD: 0.61; 95% CI: 0.49, 0.73) were better than placebo. In the network meta-analysis, the MMSE outcome ranked music therapy (59%) as the best and Acupuncture (26%) as second. Nutrition and Ginkgo treatment had the lowest rank among all interventions. For ADAS-cog outcome, acupuncture (52) ranked the best.

**Conclusion:** Among the nine treatments studied, music therapy appears to be the best treatment for MCI, followed by acupuncture. Our study provides new insights into potential clinical treatments for MCI due to AD, and may aid the development of guidelines for MCI in AD.

## Introduction

Mild cognitive impairment (MCI) has been defined as a “transitional” state to describe individuals who are not cognitively “active” for their age, but who would not meet a clinical diagnosis of early dementia (Brendan and Kelley, 2015). It is an intermediate clinical condition with a number of sub-types and multiple pathologies (Petersen, 2004; Rountree et al., 2007).

MCI is currently an area of considerable clinical and research interest because a large proportion of patients with MCI develop Alzheimer's disease (AD). In order to delay the progress of AD, it is important to recognize the key neurobiological difference between MCI and AD. Several journal articles have demonstrated that MCI patients progress to AD at a higher rate (10–15% per year) than normal elderly patients (1–3% per year). Furthermore, two-thirds of patients with AD were previously recognized as having MCI (Rubin et al., 1989; Almkvist et al., 1998; Wolf et al., 1998; Kluger et al., 1999; Petersen et al., 1999, 2001; Collie and Maruff, 2000; Morris et al., 2001). Therefore, patients who have MCI are considered to be at a greater risk for AD. Unfortunately, treatment options for AD are currently suboptimal, especially in the advanced stage of AD where the brain damage is irreversible. Thus, it is important to start treatment for AD as early as possible, and intervention during the MCI stage may prevent or delay the occurrence of AD.

Several treatments have been used for MCI, including cholinesterase inhibitors (ChEIs), complementary and alternative medicine, lifestyle and nutrition interventions, and Chinese medicine. However, which of these interventions work, and to what extent, remains unknown.

Pairwise meta-analysis have previously been conducted to assess the efficacy of ChEIs, including donepezil, galantamine, and rivastigmine (Cooper et al., 2013; Tricco et al., 2013; Matsunaga et al., 2019). These studies suggested that ChEIs have a low efficacy in the treatment of MCI and many safety issues were raised. Therefore, before using ChEIs for MCI, other methods should be considered.

Other studies (Andreas et al., 2015; Liang et al., 2018) have suggested that physical exercise and computerized cognitive training could improve cognitive function and neuropsychiatric symptoms. Furthermore, non-pharmacological therapies might perform more effectively than pharmacological therapies. However, these studies were incomplete and provide insufficient data, because they were unable to introduce clear hierarchies among treatments and a number of interventions were not analyzed. Therefore, the aim of this article was to conduct a network meta-analysis to thoroughly compare and rank different treatments for MCI that help to improve cognitive function and prevent or delay the occurrence of AD.

## Methods

### Search Strategy

Four English databases (Medline [via Ovid], Embase [via Ovid], Cochrane Library [Central Register of Controlled Trials], and Web of Science [via Ovid]) and three Chinese databases (China Science Journal Citation Report [VIP], China National Knowledge Infrastructure [CNKI], and Wanfang) were searched for all relevant citations published from the date of the respective database onset to September 31st 2019. We established search strategies which combined subject word (keyword) and random words related to MCI, interventions of interest (drug therapy, diet/lifestyle therapy, physical activity/exercise, complementary therapies, sham/placebo) and randomized controlled trial (RCT). Furthermore, the reference lists of the included studies were manually reviewed to look for additional relevant manuscripts. The specific search strategies are shown in Supplementary Datasheet 1.

### Selection and Exclusion Criteria

The RCTs which met the following criteria were included: (1) participants had mild cognitive impairment due to AD; (2) the interventions were pharmacological therapies including Cholinesterase inhibitors, Memantine, Ginkgo biloba, Huperzine A, Piracetam, Nimodipine, and Chinese medicine, or non-pharmacological therapies including acupuncture, music therapy, lifestyle therapy, exercise therapy, and nutrition therapy; (3) the comparisons were placebo, no intervention, usual care control, or other comparable interventions; (4) the study included at least one cognitive performance outcome measure in the form of either the MMSE (Mini-Mental State Examination) or ADAS-cog (AD Assessment Scale-cognitive subscale) and the efficacy of the studies must include mean changes from baseline to endpoint.

Studies with the following characteristics were excluded: (1) participants with a diagnosis of MCI due to diseases other than AD; (2) irrelevant outcomes or deficient data; and (3) case reports, review articles, clinical protocols, conference abstracts, and animal experimental studies.

### Data Extraction and Quality Assessment

Two investigators (XL and YL) screened the articles and extracted the data and related statistics independently. Basic information was organized into a standard table, including information on the characteristics of the population, intervention(s), comparison(s), treatment duration, event(s), and outcome. The Cochrane Risk of Bias Tool (Savovic et al., 2014) was used by two researchers (XL and YL) to assessed the risk of bias and quality of included trials. A third reviewer (HW) was consulted to recheck studies when the first two reviewers had disagreements and discrepancies.

### Outcomes

Our network meta-analysis used Mini-Mental State Examination (MMSE) as the primary outcome to measure global cognition, with higher MMSE scores meaning better cognitive function. The second outcome was the AD Assessment Scale-cognitive subscale (ADAS-cog), in which lower scores mean better cognitive function. If the complete data on outcomes were not reported in the original article, we contacted authors via email to obtain the raw data, otherwise studies lacking such data were eliminated. The data were independently collected by two reviewers (XL and YL) and rechecked by a third investigator (HW).

### Statistical Analysis

Firstly, a pair-wise meta-analysis was used to compare the compliance of different therapies. By using the Aggregate Data Drug Information System (ADDIS ver. 1.16.8, available at: https://drugis.org/software/addis/index) with a random-effects model, the odds ratio (OR) was measured for discontinuous outcomes along with 95% credible intervals (CI). Statistical heterogeneity was calculated in the pair-wise comparisons with an *I*^2^ statistic and the *p*-value.

Furthermore, we conducted a network meta-analysis. This network meta-analysis was set up with a Bayesian framework using the Aggregate Data Drug Information System (ADDIS ver. 1.16.8, available at: https://drugis.org/software/addis/index) to determine the cognitive outcomes of nine interventions. This software uses the Bayesian framework as a base combined with the Markov chain Monte Carlo method to evaluate research data. A random-effects model was used to evaluate the effect sizes in this network meta-analysis. Random-effect model attempted to generalize findings beyond the included studies by assuming that the selected studies are random samples from a larger population (Cheung et al., 2012). The mean difference (MD) was the effect size for continuous outcomes. The models used to estimate the effect size in ADDIS were “consistency” and “inconsistency.” The consistency model aims to evaluate effect sizes of the interventions, which was used to calculate the ranking probabilities for the whole group of interventions. Node-splitting analysis helps to determine the consistency test with an inconsistency model. For instance, a consistency model was chosen when the *p*-value of the node-splitting analysis was >0.05. If the *p*-value of the node-splitting analysis was <0.05, an inconsistency model was selected. In order to evaluate the convergence of the model, the potential scale reduction factor (PSRF) was used. If the PSRF value was close to 1, the convergence of the model was more desirable. If the PSRF value was <1.2, it was still considered as acceptable. For each intervention, the ranking probabilities were estimated for every treatment at every possible rank.

## Results

### Study Identification and Selection

**Figure 1:**
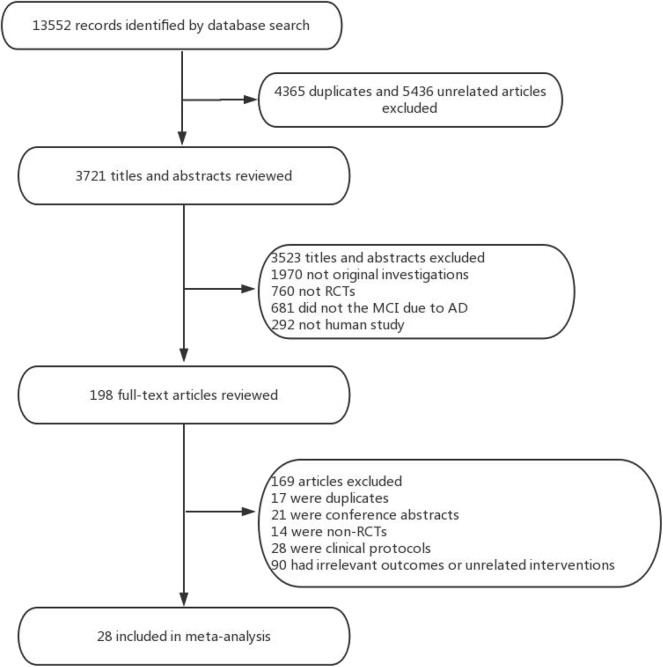
Study selection. RCT, randomized controlled trial.

Overall, a total of 13,522 studies were identified from the seven electronic databases using the search strategy, and 198 relevant full-text articles were evaluated for eligibility. There were 169 citations excluded, including 17 duplicates, 21 conference abstracts, 28 clinical protocols, 14 non-RCTs, and 90 unrelated intervention or outcome articles. Ultimately, 28 studies including 6,863 patients were clinical eligible to be included in this network meta-analysis (Figure 1). The characteristics of the selected studies were listed in Table 1.

**Table 1:** Characteristics of included studies.

**References**	**Country**	**Number of people (*n*)**	**Age (Y)**	**Intervention**	**Comparison**	**Treatment duration**	**Events**	**Outcomes**
Doody et al. (2009)	USA	DON: 409 PLA: 412	55–90	Donepezil (5–10 mg)	Placebo	48 w	1. Diarrhea (donepezil: 16.4%; placebo: 3.4%) 2. Muscle spasms (donepezil: 13.3%; placebo: 1.8%) 3. Insomnia (donepezil: 8.2%; placebo: 4.4%).	Primary: MMSE; Secondary: modified ADAS-cog
Salloway et al. (2004)	USA	DON: 133 PLA: 137	55–90	Donepezil (5–10 mg); Placebo	Placebo	24 w	1. Diarrhea (donepezil: 27%; placebo: 10%) 2. Abnormal dreams (donepezil 23%; placebo: 4%), 3. insomnia (donepezil: 11%; placebo 5%).	Modified ADAS-cog
Winblad et al. (2008)	USA, Canada	GAL: 494 PLA: 496	≥50	Galantamine (16–24 mg); Placebo	Placebo	24 m	1. Nausea (GAL: 29%; PAL: 10%) 2. Diarrhea (GAL: 15%; PAL: 9%) 3. Insomnia (GAL: 10%; PAL: 7%)	Modified ADAS-cog; ADCS-ADL
Winblad et al. (2008)	USA, Canada	GAL: 532 PLA: 526	≥50	Galantamine (16–24 mg);	Placebo	24 m	1. Nausea (GAL: 29%; PAL: 10%) 2. Diarrhea (GAL: 15%; PAL: 9%) 3. Insomnia (GAL: 10%; PAL: 7%)	Modified ADAS-cog; ADCS-ADL
Wilkinson et al. (2002)	UK, South Africa, Switzerland	RIV: 55 Don: 56	≥50	Rivastigmine (12 mg)	Donepezil (5–10 mg)	12 w	1. Nausea (RIV: 41.8%; DON: 10.7%) 2. Vomiting (RIV: 23.6%; DON: 7.1%) 3. Headache (RIV: 18.2%; DON: 7.1%)	Primary: MMSE Secondary: ADAS-cog
Howard and Roger (2007)	Australia, Canada, Italy, South Africa, UK	RIV: 227 PLA: 222	≥50	Rivastigmine (6–36 mg)	Placebo	26 w	1. Nausea (RIV: 48.0%; PLA: 14.0%) 2. Vomiting (RIV: 30.0%; PLA: 6.3%) 3. Anorexia (RIV: 18.5%; PLA: 2.4%)	Primary: MMSE; Secondary: ADAS-cog
Dong et al. (2012)	China	GIN: 58 PLA: 55	60–85	Ginkgo glycosides (9.6 mg tid)	Placebo	1 y	Not mentioned	MMSE
Xiao et al. (2011)	China	GIN: 54 PLA: 44	55–85	Ginkgo glycosides (19.2 mg tid)	Placebo	6 m	Not mentioned	MMSE
Aisen et al. (2008)	USA	VB: 240 PLA: 169	≥50	Folate: 5 mg, Vitamin B6: 25 mg, Vitamin B12: 1 mg	Placebo	18 m	1. Depression (VB: 27.9%; PAL: 17.8%) 2. Restlessness (VB: 12.1%; PAL: 8.3%) 3. Hyperhidrosis (VB: 9.6%; PAL: 4.1%)	Primary: MMSE; Secondary: ADAS-cog
Petersen et al. (2005)	USA, Canada	VE: 257 PLA: 259	55–90	Vitamin E: 2,000 IU	Placebo	36 m	1. Diarrhea (VE: 10.2%; placebo: 6.6%) 2 Insomnia (VE: 3.1%; placebo: 1.9%) 3. Nausea (VE: 1.2%; placebo: 1.9%).	Primary: MMSE Secondary: ADAS-cog
Sun et al. (2007)	Taiwan	Multivitamin: 45 PLA: 44	≥50	Multivitamin supplement^*^+ Mecobalamin: 0.5 mg	Placebo	26 w	1. Muscle pain (multivitamin: 11.1%; PAL: 6.8%) 2. Insomnia (multivitamin: 8.9%; PAL: 9.1%) 3. Delirium (multivitamin: 8.9%; PAL: 2.3%)	Primary: MMSE Secondary: ADAS-cog
Dysken et al. (2014)	/	VE: 152 PLA: 152	60–75	2,000 IU vitamin E	Placebo	4 y	Not mentioned	Primary: MMSE Secondary: ADAS-cog
Tian et al. (2019)	China	CM: 174 PLA: 70	≥50	Qinggongshoutao 27 g; EGb761 160 mg	Placebo	52 w	Upper respiratory tract infection, diarrhea, constipation, urinary tract infection, and increased blood glucose	Primary: MMSE Secondary: ADAS-cog
Zhou et al. (2007)	China	CM: 42 PLA: 37	53–79	Shenyin Oral Liquid	Placebo	12 m	Not mentioned	MMSE
Wei et al. (2012)	China	CM: 24 PLA: 23	45–80	Danshen, Sanqi	Placebo	12 w	CM: stomachache (2) PLA: urinary tract infection (2)	Primary: MMSE Secondary: ADAS-cog
Miao et al. (2012)	China	CM: 45 PLA: 48	40–85	CM; 200 ml	Placebo	12 w	Not mentioned	Primary: MMSE Secondary: ADAS-cog
Suzuki et al. (2013)	Japan	Exercise: 50 PLA: 50	≥65	Multicomponent exercise (bi-weekly 90-min): aerobic exercise, muscle strength training, postural balance retraining, and dual-task training	Placebo	6 m	Not mentioned	Primary: MMSE Secondary: ADAS-cog
Lautenschlager et al. (2008)	Australia	Exercise: 48 PLA: 52	≥50	≥150 min of moderate-intensity physical activity per week	Placebo	18 w	1. Cardiovascular problems (EXE: 6.3%; PLA: 1.9%) 2. Stroke or transient ischemic attack (EXE: 2.1%; PLA: 1.9%)	ADAS-cog
Grace et al. (2013)	Hong Kong	Exercise: 7 PLA: 6	≥60	Computer-assisted EL memory training; Therapist-led EL memory training group	Placebo	3 m	Not mentioned	MMSE
Tsai et al. (2013)	USA	Taichi: 28 PLA: 27	≥60	Taichi (3 sessions a week)	Placebo: attention control group	20 w	Not mentioned	MMSE
Doi et al. (2017)	Japan	Music: 67 Con: 67	≥70	Music: playing percussion instruments at weekly 60-min sessions	Control: health education classes	40 w	No injuries reported	MMSE
Li et al. (2015)	Taiwan	Music: 20 PLC: 21	≥60	Music: Mozart's Sonata (KV 448) and Pachelbel's Canon, listening with headphones for 30 min daily in the morning and before sleep	Placebo	6 m	Not mentioned	MMSE
Cai et al. (2019)	China	Music: 25 PLC: 25	60–74	Music: 1–1.5 h music therapy, 3 times a weeks	Placebo	3 m	Not mentioned	MMSE
Yang et al. (2019)	China	ACU: 108 PLC: 105	55–85	Acupuncture: 30 min twice a week (at an interval of 2–4 days)	Placebo	6 m	Not mentioned	ADAS-cog
Jia et al. (2017)	China	ACU: 43 ChEIs: 44	50–85	Acupuncture: 30 min, 3 times weekly	Donepezil: 5–10 mg	28 w	Not mentioned	ADAS-cog
Zhu et al. (2015)	China	ACU: 30 NIM: 30	46–75	Acupuncture: Acupuncture and Moxibustion (30 min each, 6 times weekly)	Nimodipine 30 mg, 3 times a day	8 w	Not mentioned	MMSE
Chen (2011)	China	ACU: 80 PLC: 75	55–85	Acupuncture: Acupuncture (30 min, 3 times weekly)	Placebo	8 w	Acu: fainting during acupuncture treatment 10 Drug: gastrointestinal reaction 9	MMSE
Zheng et al. (2008)	China	CM: 30 NIM: 30	/	Nimodipine 30 mg, 3 times a day	Chinese medicine: once a day	12 w	Not mentioned	MMSE

* *Containing: iron ferrous 60 mg, nicotinamide 10 mg, calcium carbonate 250 mg, riboflavin 2 mg, thiamine mononitrate 3 mg, calcium pantothenate 1 mg, ascorbic acid 100 μg, iodine 100 μg, copper 150 μg, vitamin B12 3 μg, vitamin A 4,000 IU, and vitamin D3 400 IU*.

*ACU, acupuncture; ChEIs, cholinesterase inhibitors; CM, Chinese medicine; Con, control; d, days; m, months; NIM, nimodipine; PLC, placebo; w, weeks; y, years*.

### Study Quality

**Figure 2:**
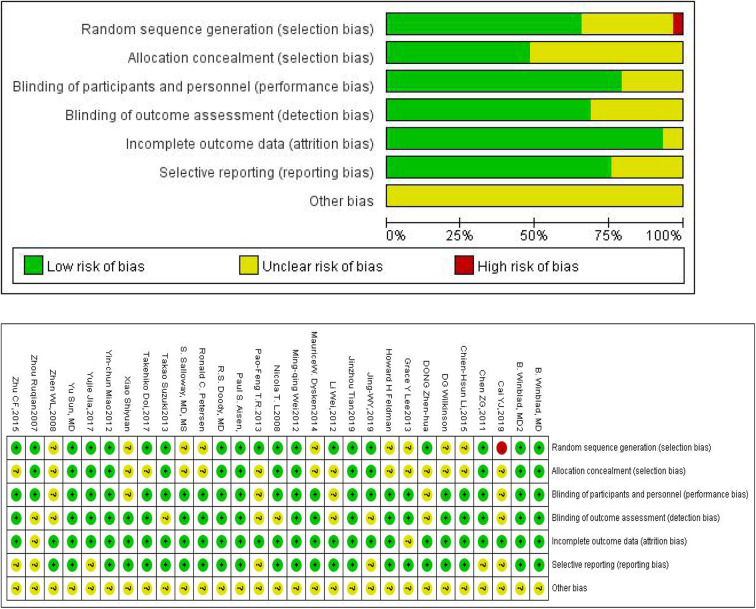
Quality assessment of included studies.

The Cochrane Risk of Bias Tool was used to assess the quality of all 28 trials included in our study (Figure 2 shows the summary risk of bias for selected studies). Among the 28 trials, 19 (66%) represented a random sequence generation process using a computer random number generator or a random number table. Fourteen trials (48.3%) described the use of allocation concealment methods, and 22 trials (75.9%) described the blinding methods for researchers and participants. Acupuncture, music therapy, and exercise therapy are non-pharmacologic therapies, therefore some participants and researchers involved in these studies were not able to be blinded. Six trials (21.4%) had an uncertain risk of outcome assessment and 25 trials (86.2%) had a low risk of attrition bias.

### Pair-Wise Meta-Analysis

We conducted a classic pair-wise meta-analysis using a random-effects model to synthesize studies with the same pair of interventions. All interventions, except for nimodipine, had at least one placebo controlled trial. As for the MMSE outcome, the ChEIs (MD: −0.38; 95% CI: −0.74, −0.01), Chinese medicine (MD: −0.31; 95% CI: −0.75, 0.13), exercise therapy (MD: −0.50; 95% CI: −0.65, −0.35), music therapy (MD: −1.71; 95% CI: −4.49, 1.07), were worse than placebo,and other interventions were statistically more efficient than placebo. For ADAS-cog outcome, ChEIs (MD: 1.20; 95% CI: 0.73, 1.68), Acupuncture (MD: 1.36; 95% CI: 1.28, 1.44), Chinese medicine (MD: 0.61; 95% CI: 0.49, 0.73) and exercise (MD: 0.61; 95% CI: 0.49, 0.73) were better than placebo. The detailed results of the pair-wise meta-analysis are shown in Supplementary Datasheet 2.

### Network Meta-Analysis

#### Primary Outcome: MMSE

**Figure 3:**
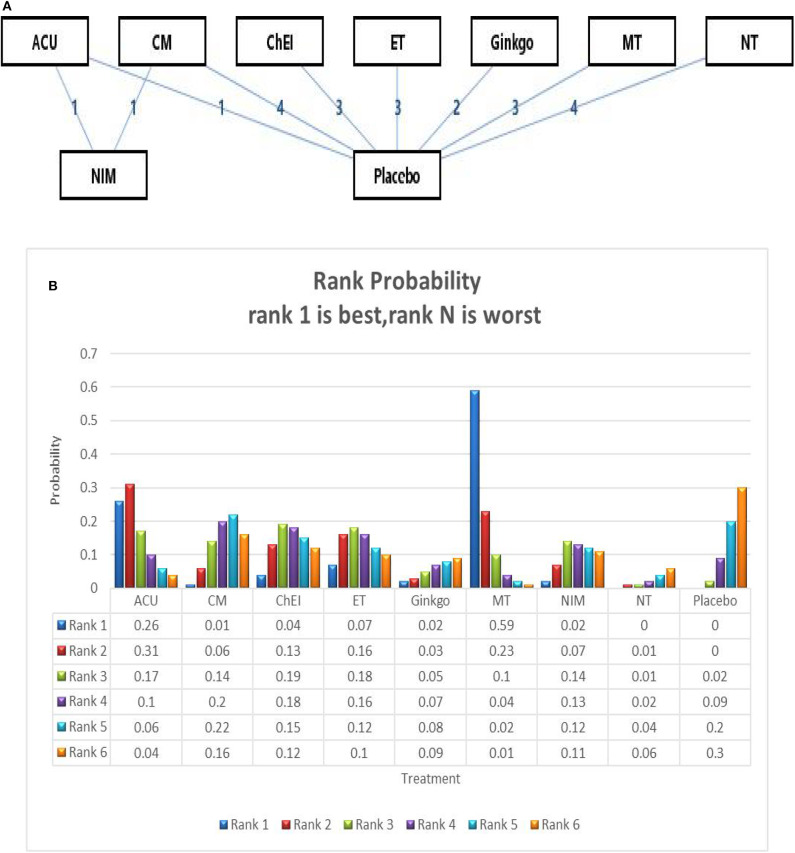
**(A)** The network structure of the analyzed treatment comparisons for the outcome of MMSE. **(B)** Rank probability of cognitive effects of MMSE. ACU, acupuncture; CM, Chinese medicine; CHEI, cholinesterase inhibitors; ET, exercise therapy; MT, music therapy; NIM, nimodipine; NT, nutrition therapy.

We ran a network meta-analysis to thoroughly compare and assess different treatment rankings for MCI, the network of MMSE include 23 trails and 15 interventions,the network plot presented in Figure 3A. Node-splitting analysis was used to assess consistency, and all *p*-values between the direct and indirect effects were > 0.05. A PSRF value of 1 indicated that the model was convergent and the result was stable. Therefore, the consistency model was selected for the subsequent network analysis.

The network meta-analysis for the primary outcome (MMSE) is shown in Table 2. In terms of efficacy music therapy (*MD*: 1.74; 95% CI: 0.21, 3.26), Acupuncture (*MD*: 1.22; 95% CI:−0.97, 3.39), and exercise therapy (*MD*: 0.52; 95% CI: −1.22, 2.28) achieved better than placebo. Ginkgo (*MD*: −0.40; 95% CI: −2.34, 1.57) and nutrition therapy (*MD*: −0.75; 95% CI: −2.04, 0.61) were significantly less effective than other interventions and placebo. Other pharmacological therapies including Chinese medicine (*MD*: 0.27; 95% CI: −0.96, 1.52) and ChEIs (*MD*: 0.46; 95% CI: −1.02, 1.96) showed a slight improvement in MMSE scores; however, their efficacy in MCI needs further investigation. The ranking probability of MMSE is presented in Figure 3B, the results showed that music therapy had the highest probability (59%) of being the best treatment for MCI, followed by Acupuncture (26%) and then exercise (7%).

**Table 2:** The consistency model of MMSE, comparisons should be read from left to right.

ACU	−0.93 (−3.25, 1.38)	−0.76 (−3.44, 1.92)	−0.70 (−3.47, 2.19)	−1.62 (−4.51, 1.26)	0.52 (−2.13, 3.19)	−1.18 (−3.38, 1.02)	−1.95 (−4.51, 0.59)	−1.22 (−3.39, 0.97)
0.93 (−1.38, 3.25)	CM	0.19 (−1.74, 2.13)	0.25 (−1.86, 2.35)	−0.68 (−2.95, 1.65)	1.45 (−0.50, 3.44)	−0.22 (−2.46, 1.96)	−1.02 (−2.79, 0.77)	−0.27 (−1.52, 0.96)
0.76 (−1.92, 3.44)	−0.19 (−2.13, 1.74)	ChEI	0.07 (−2.25, 2.33)	−0.86 (−3.35, 1.60)	1.26 (−0.82, 3.40)	−0.43 (−3.19, 2.34)	−1.19 (−3.13, 0.82)	−0.46 (−1.96, 1.02)
0.70 (−2.19, 3.47)	−0.25 (−2.35, 1.86)	−0.07 (−2.33, 2.25)	ET	−0.93 (−3.51, 1.69)	1.22 (−1.12, 3.47)	−0.48 (−3.40, 2.37)	−1.27 (−3.41, 0.95)	−0.52 (−2.28, 1.22)
1.62 (−1.26, 4.51)	0.68 (−1.65, 2.95)	0.86 (−1.60, 3.35)	0.93 (−1.69, 3.51)	Ginkgo	2.14 (−0.34, 4.51)	0.47 (−2.58, 3.40)	−0.33 (−2.67, 2.06)	0.40 (−1.57, 2.34)
−0.52 (−3.19, 2.13)	−1.45 (−3.44, 0.50)	−1.26 (−3.40, 0.82)	−1.22 (−3.47, 1.12)	−2.14 (−4.51, 0.34)	MT	−1.71 (−4.43, 1.08)	−2.48 (−4.45, −0.41)	−1.74 (−3.26, −0.21)
1.18 (−1.02, 3.38)	0.22 (−1.96, 2.46)	0.43 (−2.34, 3.19)	0.48 (−2.37, 3.40)	−0.47 (−3.40, 2.58)	1.71 (−1.08, 4.43)	NIM	−0.77 (−3.42, 1.88)	−0.03 (−2.35, 2.30)
1.95 (−0.59, 4.51)	1.02 (−0.77, 2.79)	1.19 (−0.82, 3.13)	1.27 (−0.95, 3.41)	0.33 (−2.06, 2.67)	2.48 (0.41, 4.45)	0.77 (−1.88, 3.42)	NT	0.75 (−0.61, 2.04)
1.22 (−0.97, 3.39)	0.27 (−0.96, 1.52)	0.46 (−1.02, 1.96)	0.52 (−1.22, 2.28)	−0.40 (−2.34, 1.57)	1.74 (0.21, 3.26)	0.03 (−2.30, 2.35)	−0.75 (−2.04, 0.61)	Placebo

*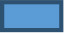 Treatments; 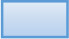 Efficacy (mean overall change, SMD [95% Crl])*.

#### Secondary Outcome: ADAS-cog

**Figure 4:**
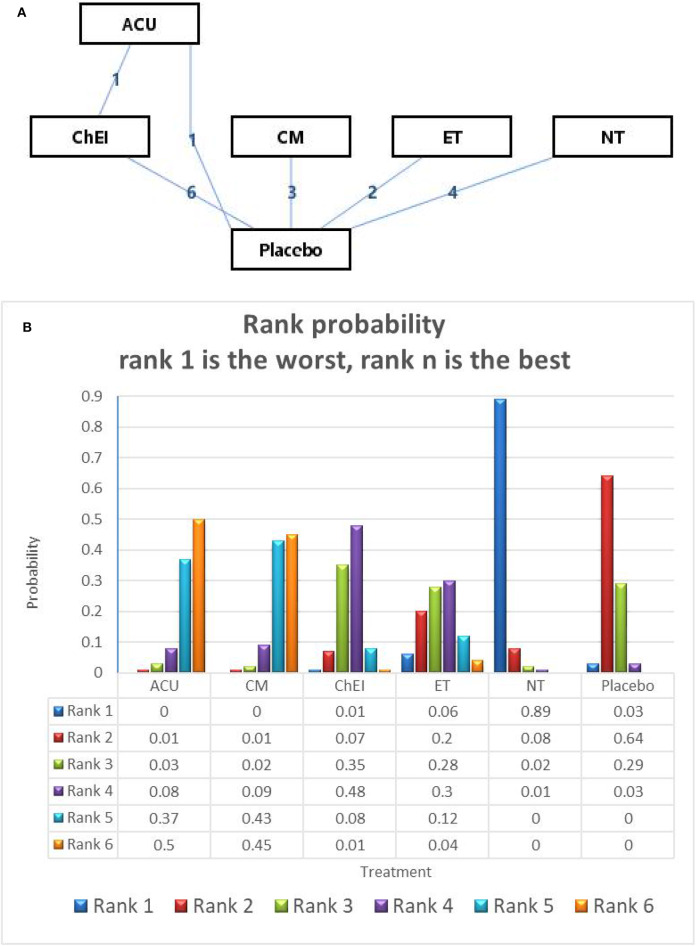
**(A)** The network structure of the analyzed treatment comparisons for the outcome of ADAS-cog. **(B)** Rank probability of cognitive effects of ADAS-cog.

In relation to the secondary outcome (ADAS-cog score), 17 trails and 6 treatments were involved, 2 arms of Acupuncture, 6 arms of ChEIs, 3 arms of Chinese medicine, 2 arms of exercise therapy and 4 arms of nutrition therapy (presented in Figure 4A). After Node-splitting analysis,we adopted the consistency model to compare these different interventions. Chinese medicine (*MD*: −2.72; 95% CI: −4.83, −0.56), Acupuncture (*MD*: −2.84; 95% CI: −5.61, −0.34), exercise therapy (*MD*: −0.71; 95% CI: −3.12, 1.68), and ChEIs (*MD*: −0.88; 95% CI: −2.23, 0.53) were better than placebo. Nutrition therapy (*MD*: 1.43; 95% CI: −0.34, 3.14) was least effective compared to other interventions and placebo (shown in Table 3). The ranking probability of ADAS-cog is presented in Figure 3B. Acupuncture ranked the best (52%), it might be the most effective way to change the ADAS-cog score.

**Table 3:** The consistency model of ADAS-cog, comparisons should be read from left to right.

ACU	0.10 (−3.17, 3.61)	1.97 (−0.65, 4.76)	2.14 (−1.35, 5.88)	4.26 (1.28, 7.53)	2.84 (0.34, 5.61)
−0.10 (−3.61, 3.17)	CM	1.85 (−0.61, 4.35)	2.02 (−1.23, 5.16)	4.15 (1.38, 6.87)	2.72 (0.56, 4.83)
−1.97 (−4.76, 0.65)	−1.85 (−4.35, 0.61)	ChEI	0.16 (−2.61, 2.94)	2.30 (0.04, 4.51)	0.88 (−0.53, 2.23)
−2.14 (−5.88, 1.35)	−2.02 (−5.16, 1.23)	−0.16 (−2.94, 2.61)	ET	2.13 (−0.83, 5.09)	0.71 (−1.68, 3.12)
−4.26 (−7.53, −1.28)	−4.15 (−6.87, −1.38)	−2.30 (−4.51, −0.04)	−2.13 (−5.09, 0.83)	NT	−1.43 (−3.14, 0.34)
−2.84 (−5.61, −0.34)	−2.72 (−4.83, −0.56)	−0.88 (−2.23, 0.53)	−0.71 (−3.12, 1.68)	1.43 (−0.34, 3.14)	Placebo

*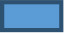 Treatments, 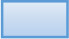 efficacy (mean overall change, SMD [95% Crl])*.

### Adverse Events

Out of 28 trials, 13 trails reported adverse events occurred, 14 trails did not mention whether there was adverse events and one trial stated no injures reported (Table 1). 9 out of 13 trials reported adverse events related to gastrointestinal discomforts such as diarrhea, nausea, vomiting and anorexia, these interventions were mainly ChEIs, nutrition therapy and Chinese medicine. 8 out of 13 trails reported insomnia as adverse effect which mainly related to ChEIs and nutrition interventions. 1 out of 13 trails reported cardiovascular problem due to exercises intervention and 1 out of 13 trials reported Acupuncture-fainting due to Acupuncture intervention.

## Discussion

This study is the first network meta-analysis performed on potential pharmacological and non-pharmacological treatments for MCI, and has incorporated the most comprehensive data. The Bayesian statistical methods that we used allowed us to rank many different treatments by measuring comparable probability and then reporting them as the best, the second best, and so on. Through this method, we found that among all interventions, both pharmacological therapies (ChEIs, ginkgo, nimodipine, and Chinese medicine) and non-pharmacological therapies (acupuncture, music therapy, exercise, and nutrition therapy), music therapy and acupuncture were better than other treatments. Some pharmacological treatments like CHEIs such as donepezil, galantamine, and rivastigmine showed a slight efficacy to improve MMSE scores and cognitive function, but have many safety issues which require further clarification and study. In addition, ginkgo and nutrition therapy might be ineffective for MCI, which are thus not strongly recommended in clinical medicine.

Music therapy is one of the non-pharmacological therapies which incorporates active and passive therapy. Active music therapy can be defined as therapeutic music activities which involve active participation by clients, such as singing, dancing with music, playing an instrument, composing, and discussing clients' thoughts and feelings in regards to music-related activities in order to reach the optimal treatment effect. Passive music therapy, on the other hand, means achieving the treatment effect by listening and enjoying music. Passive music therapy is also called receptive music therapy and is widely used (Chang et al., 2015; Han et al., 2017; Claudia et al., 2019). Previous research has shown that music can activate some brain regions that govern cognitive function, affective function, and motor skills, and activate neurological stimulation which may develop new neural networks (Raglio et al., 2014; Mofredj et al., 2016). Evidence has shown that, in the context of patients with cognitive function decline, music therapy intervention increases cerebral blood flow and pre-frontal cortex activity (Shimizu et al., 2018).

Acupuncture has been shown to be a good management option in neurological diseases, with studies demonstrating acupuncture is associated with activation in motor-related brain regions as shown in functional magnetic resonance imaging (fMRI) (Chen et al., 2008, 2012; Chae et al., 2009). Other studies supplemented that Acupuncture is able to regulate the emotional components of the pain matrix, and inducing brain activation which provides a neurobiological basis of acupuncture (Shangjie et al., 2014; Shan et al., 2018). Research also suggests that acupuncture can activate resting brain networks, which incorporate anti-nociceptive, memory, and emotion brain regions. Our network meta-analysis suggests that music therapy is the optimal intervention to treat MCI patients and improve their cognitive function, whereas acupuncture was the second best option.Further research is required to assess other interventions not included in this meta-analysis. A recent study found that mindfulness therapy improved inflammatory biomarkers in patients with MCI (Ng et al., 2020). This provides an interesting perspective on the current management paradigms for MCI.

The adverse events reports showed that some participants experienced gastrointestinal reactions and insomnia due to ChEIs, nutrition and Chinese medicine interventions. However, none of these events was related to cognitive distress. Other small samples such as exercise and Acupuncture reported therapy related adverse effects such as cardiovascular problems and Acupuncture fainting respectively. Music therapy didn't report any adverse events.

There were limitations to our study. Firstly, a few RCTs showed potential bias because of the small number of participants and elective reporting. Fortunately, there was no obvious inconsistency or heterogeneity shown in this network meta-analysis, but there is a possibility that some included articles might have overestimated the effectiveness of treatments, and this might have influenced our results. Secondly, a few included reports were non-pharmaceutical therapies which cannot be blinded to participants, especially acupuncture. However, blinding of outcome assessment and single-blind methodologies should be used where possible to reduce the potential for any bias. Thirdly, we have excluded some drug interventions because of our selection criterion of outcome measures, which may influence the strength of evidence.

## Conclusion

The findings of this comprehensive network meta-analysis provide some evidence that music therapy and acupuncture might improve the cognitive function of patients with MCI. Our results indicate that music therapy and, to a lesser extent, acupuncture may be the preferred options for treatment of MCI. Ginkgo and nutrition therapy do not seem to be adequate as regular treatment options. Our study provides new insights into the clinical treatments available for MCI, and may help the development of guidelines for the management of MCI.

## Data Availability Statement

All datasets generated for this study are included in the article/Supplementary Material.

## Author Contributions

CT and LL designed the study. XL, YL, and HW collected the data. XL performed all analysis. XL, YL, and HW wrote the manuscript. All authors contributed to writing of this manuscript.

## Conflict of Interest

The authors declare that the research was conducted in the absence of any commercial or financial relationships that could be construed as a potential conflict of interest.
