# Supplementary material for: The Comparative Efficacy of Multiple Interventions for Mild Cognitive Impairment in Alzheimer's Disease: A Bayesian Network Meta-Analysis
Source: Front Aging Neurosci. 2020 Jun 5;12:121. doi: 10.3389/fnagi.2020.00121 (PMC7289916; doi:10.3389/fnagi.2020.00121)
Supplement: Supplementary file 1 [file Data_Sheet_1.PDF]

Search strategy (DR.Lai Xin)

MEDLINE (OVID) search strategy

1. exp Mild cognitive impairment
2. (Mild cognitive impairment OR Cognitive Dysfunctions OR Dysfunctions, Cognitive OR Cognitive Impairments OR Cognitive Impairment OR Impairment, Cognitive OR Impairments, Cognitive OR Mild Cognitive Impairment OR Cognitive Impairment, Mild OR Cognitive Impairments, Mild OR Impairment, Mild Cognitive OR Impairments, Mild Cognitive OR Mild Cognitive Impairments OR Mild Neurocognitive Disorder OR Disorder, Mild Neurocognitive OR Disorders, Mild Neurocognitive OR Mild Neurocognitive Disorders OR Neurocognitive Disorder, Mild OR Neurocognitive Disorders, Mild OR Cognitive Decline OR Cognitive Declines OR Decline, Cognitive OR Declines, Cognitive OR Mental Deterioration OR Deterioration, Mental OR Deteriorations, Mental OR Mental Deteriorations) .mp.
3. 1 OR 2
4. exp Drug Therapy
5. (Therapy, Drug OR Drug Therapies OR Therapies, Drug OR Chemotherapy OR Chemotherapies OR Pharmacotherapy OR Pharmacotherapies OR Inhibitors, Cholinesterase OR Anticholinesterase Drugs OR Drugs, Anticholinesterase OR Anticholinesterases OR Anticholinesterase Agents OR Agents, Anticholinesterase OR Anti-Cholinesterases OR Anti Cholinesterases OR Cholinesterase Inhibitors, Irreversible OR Inhibitors, Irreversible Cholinesterase OR Irreversible Cholinesterase Inhibitors OR Cholinesterase Inhibitors, Reversible OR Inhibitors, Reversible Cholinesterase OR Reversible Cholinesterase Inhibitors OR Acetylcholinesterase Inhibitors OR Inhibitors, Acetylcholinesterase OR Memantine OR donepezil OR rivastigmine OR galantamine).mp.
6. 4 OR 5
7. Exp diet, lifestyle therapy
8. (nutritional management OR dietary management OR Mediterranean Diet OR Diets, Mediterranean OR Mediterranean Diets OR Mediterranean-style lifestyle OR lifestyle OR life style OR lifestyles OR Social engagement OR social work OR social works).mp.
9. 7 OR 8

10.exp physical activity

11.(exercise OR Exercises OR Physical Activity OR Activities, Physical OR Activity, Physical OR Physical Activities OR Exercise, Physical OR Exercises, Physical OR Physical Exercise OR Physical Exercises OR Acute Exercise OR Acute Exercises OR Exercise, Acute OR Exercises, Acute OR Exercise, Isometric OR Exercises, Isometric OR Isometric Exercises OR Isometric Exercise OR Exercise, Aerobic OR Aerobic Exercise OR Aerobic Exercises OR Exercises, Aerobic OR Exercise Training OR Exercise Trainings OR Training, Exercise OR Trainings, Exercise, social management).mp.

12.10 OR 11

13. exp Complementary Therapies
14. (Therapies, Complementary OR Therapy, Complementary OR Complementary Medicine OR Medicine, Complementary OR Alternative Medicine OR Medicine, Alternative OR Alternative Therapies OR Therapies, Alternative OR Therapy, Alternative OR Ginkgo bilobas OR bilobas, Ginkgo OR Gingko OR Gingkos OR Gingko biloba OR Gingko bilobas OR bilobas, Gingko OR

Ginkgo OR Ginkgos OR Ginko OR Ginkos OR Maidenhair Tree OR Maidenhair Trees OR Tree, Maidenhair OR Trees, Maidenhair OR Ginkgophyta OR Ginkgophytas OR Huperzine A OR Piracetam).mp.
15. 13 OR 14
16. Randomized controlled trial.pt.
17. controlled clinical trial.pt.
18. randomized.ti,ab.
19. placebo.ti,ab.
20. randomly.ti,ab.
21. trial.ti,ab.
22. groups.ti,ab.
23. OR/16-23
24. 6 OR 9 OR 12 OR 15
25. 3 AND 23 AND 24

EMBASE (OVID) search strategy
1. exp Mild cognitive impairment
2. (Mild cognitive impairment OR Cognitive Dysfunctions OR Dysfunctions, Cognitive OR Cognitive Impairments OR Cognitive Impairment OR Impairment, Cognitive OR Impairments, Cognitive OR Mild Cognitive Impairment OR Cognitive Impairment, Mild OR Cognitive Impairments, Mild OR Impairment, Mild Cognitive OR Impairments, Mild Cognitive OR Mild Cognitive Impairments OR Mild Neurocognitive Disorder OR Disorder, Mild Neurocognitive OR Disorders, Mild Neurocognitive OR Mild Neurocognitive Disorders OR Neurocognitive Disorder, Mild OR Neurocognitive Disorders, Mild OR Cognitive Decline OR Cognitive Declines OR Decline, Cognitive OR Declines, Cognitive OR Mental Deterioration OR Deterioration, Mental OR Deteriorations, Mental OR Mental Deteriorations) .tw.
3. 1 OR 2
4. exp Drug Therapy
5. (Therapy, Drug OR Drug Therapies OR Therapies, Drug OR Chemotherapy OR Chemotherapies OR Pharmacotherapy OR Pharmacotherapies OR Inhibitors, Cholinesterase OR Anticholinesterase Drugs OR Drugs, Anticholinesterase OR Anticholinesterases OR Anticholinesterase Agents OR Agents, Anticholinesterase OR Anti-Cholinesterases OR Anti Cholinesterases OR Cholinesterase Inhibitors, Irreversible OR Inhibitors, Irreversible Cholinesterase OR Irreversible Cholinesterase Inhibitors OR Cholinesterase Inhibitors, Reversible OR Inhibitors, Reversible Cholinesterase OR Reversible Cholinesterase Inhibitors OR Acetylcholinesterase Inhibitors OR Inhibitors, Acetylcholinesterase OR Memantine OR donepezil OR rivastigmine OR galantamine).tw.
6. 4 OR 5
7. Exp diet, lifestyle therapy
8. (nutritional management OR dietary management OR Mediterranean Diet OR Diets, Mediterranean OR Mediterranean Diets OR Mediterranean-style diet OR lifestyle life style OR lifestyles Social engagement OR social work OR social works).tw.
9. 7 OR 8
10.exp physical activity

11.(exercise OR Exercises OR Physical Activity OR Activities, Physical OR Activity, Physical OR Physical Activities OR Exercise, Physical OR Exercises, Physical OR Physical Exercise OR Physical Exercises OR Acute Exercise OR Acute Exercises OR Exercise, Acute OR Exercises, Acute OR Exercise, Isometric OR Exercises, Isometric OR Isometric Exercises OR Isometric Exercise OR Exercise, Aerobic OR Aerobic Exercise OR Aerobic Exercises OR Exercises, Aerobic OR Exercise Training OR Exercise Trainings OR Training, Exercise OR Trainings, Exercise, social management).tw.
12.10 OR 11
13. exp Complementary Therapies.mp.
14. (Therapies, Complementary OR Therapy, Complementary OR Complementary Medicine OR Medicine, Complementary OR Alternative Medicine OR Medicine, Alternative OR Alternative Therapies OR Therapies, Alternative OR Therapy, Alternative OR Ginkgo bilobas OR bilobas, Ginkgo OR Gingko OR Gingkos OR Gingko biloba OR Gingko bilobas OR bilobas, Gingko OR Ginkgo OR Ginkgos OR Ginko OR Ginkos OR Maidenhair Tree OR Maidenhair Trees OR Tree, Maidenhair OR Trees, Maidenhair OR Ginkgophyta OR Ginkgophytas OR Huperzine A OR Piracetam).tw.
15. 13 OR 14
16. Randomized controlled trial.pt.
17. controlled clinical trial.pt.
18. randomized.ti,ab.
19. placebo.ti,ab.
20. randomly.ti,ab.
21. trial.ti,ab.
22. groups.ti,ab.
23. OR/16-23
24. 6 OR 9 OR 12 OR 15
25. 3 AND 23 AND 24

CENTRAL (Cochrane Library) search strategy
1. MeSH descriptor: [Mild cognitive impairment] explode all trees
2. (Mild cognitive impairment OR Cognitive Dysfunctions OR Dysfunctions, Cognitive OR Cognitive Impairments OR Cognitive Impairment OR Impairment, Cognitive OR Impairments, Cognitive OR Mild Cognitive Impairment OR Cognitive Impairment, Mild OR Cognitive Impairments, Mild OR Impairment, Mild Cognitive OR Impairments, Mild Cognitive OR Mild Cognitive Impairments OR Mild Neurocognitive Disorder OR Disorder, Mild Neurocognitive OR Disorders, Mild Neurocognitive OR Mild Neurocognitive Disorders OR Neurocognitive Disorder, Mild OR Neurocognitive Disorders, Mild OR Cognitive Decline OR Cognitive Declines OR Decline, Cognitive OR Declines, Cognitive OR Mental Deterioration OR Deterioration, Mental OR Deteriorations, Mental OR Mental Deteriorations):ti,ab,kw (Word variations have been searched)
3.#1 or #2
4.MeSH descriptor: [Drug Therapy] explode all trees
5.(Therapy, Drug OR Drug Therapies OR Therapies, Drug OR Chemotherapy OR

Chemotherapies OR Pharmacotherapy OR Pharmacotherapies OR Inhibitors, Cholinesterase OR Anticholinesterase Drugs OR Drugs, Anticholinesterase OR Anticholinesterases OR Anticholinesterase Agents OR Agents, Anticholinesterase OR Anti-Cholinesterases OR Anti Cholinesterases OR Cholinesterase Inhibitors, Irreversible OR Inhibitors, Irreversible Cholinesterase OR Irreversible Cholinesterase Inhibitors OR Cholinesterase Inhibitors, Reversible OR Inhibitors, Reversible Cholinesterase OR Reversible Cholinesterase Inhibitors OR Acetylcholinesterase Inhibitors OR Inhibitors, Acetylcholinesterase OR Memantine OR donepezil OR rivastigmine OR galantamine) :ti,ab,kw (Word variations have been searched)

6.#4 OR #5

7.MeSH descriptor: [diet, lifestyle therapy] explode all trees

8.(nutritional management OR dietary management OR Mediterranean Diet OR Diets, Mediterranean OR Mediterranean Diets OR Mediterranean-style diet OR lifestyle life style OR lifestyles Social engagement OR social work OR social works):ti,ab,kw (Word variations have been searched)

9.#7 or #8

10.MeSH descriptor: [physical activity] explode all trees

11.(exercise OR Exercises OR Physical Activity OR Activities, Physical OR Activity, Physical OR Physical Activities OR Exercise, Physical OR Exercises, Physical OR Physical Exercise OR Physical Exercises OR Acute Exercise OR Acute Exercises OR Exercise, Acute OR Exercises, Acute OR Exercise, Isometric OR Exercises, Isometric OR Isometric Exercises OR Isometric Exercise OR Exercise, Aerobic OR Aerobic Exercise OR Aerobic Exercises OR Exercises, Aerobic OR Exercise Training OR Exercise Trainings OR Training, Exercise OR Trainings, Exercise, social management):ti,ab,kw (Word variations have been searched)

12.#10 or #11

13.MeSH descriptor: [Complementary Therapies] explode all trees

14.(Therapies, Complementary OR Therapy, Complementary OR Complementary Medicine OR Medicine, Complementary OR Alternative Medicine OR Medicine, Alternative OR Alternative Therapies OR Therapies, Alternative OR Therapy, Alternative OR Ginkgo bilobas OR bilobas, Ginkgo OR Gingko OR Gingkos OR Gingko biloba OR Gingko bilobas OR bilobas, Gingko OR Ginkgo OR Ginkgos OR Ginko OR Ginkos OR Maidenhair Tree OR Maidenhair Trees OR Tree, Maidenhair OR Trees, Maidenhair OR Ginkgophyta OR Ginkgophytas OR Huperzine A OR Piracetam):ti,ab,kw (Word variations have been searched)

15.#13 or #14

16.#6 or #9 or #12or#15

17.#3 and #16
