# Supplementary figures and images for: The Comparative Efficacy of Multiple Interventions for Mild Cognitive Impairment in Alzheimer's Disease: A Bayesian Network Meta-Analysis
Source: Front Aging Neurosci. 2020 Jun 5;12:121. doi: 10.3389/fnagi.2020.00121 (PMC7289916; doi:10.3389/fnagi.2020.00121)

## SupplementB: pair-wise analysis of MMSE

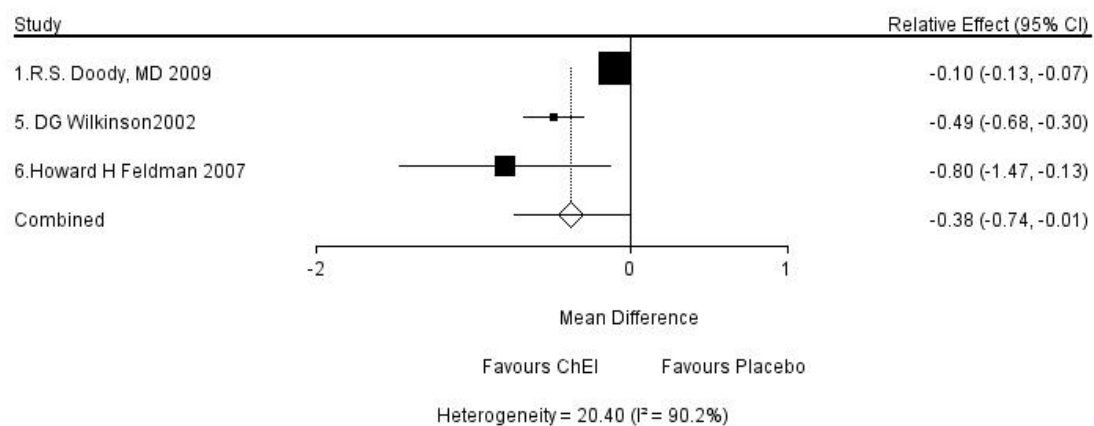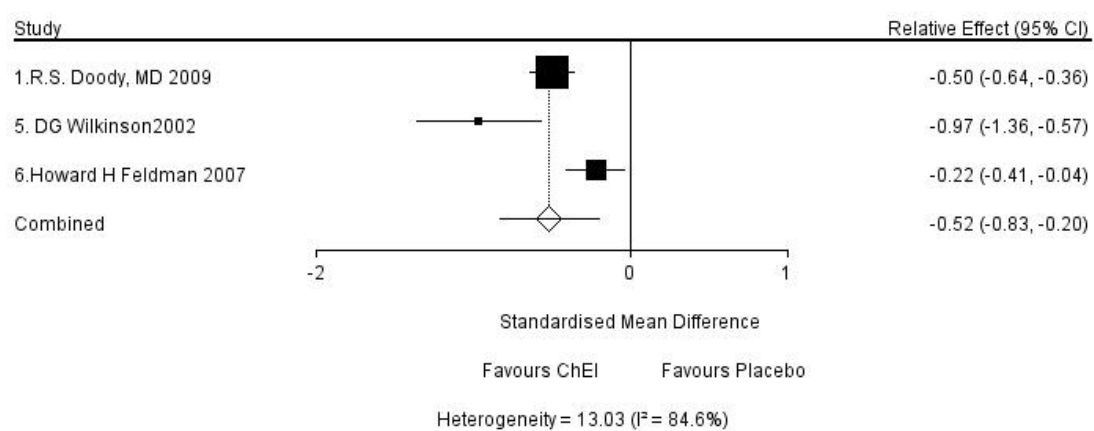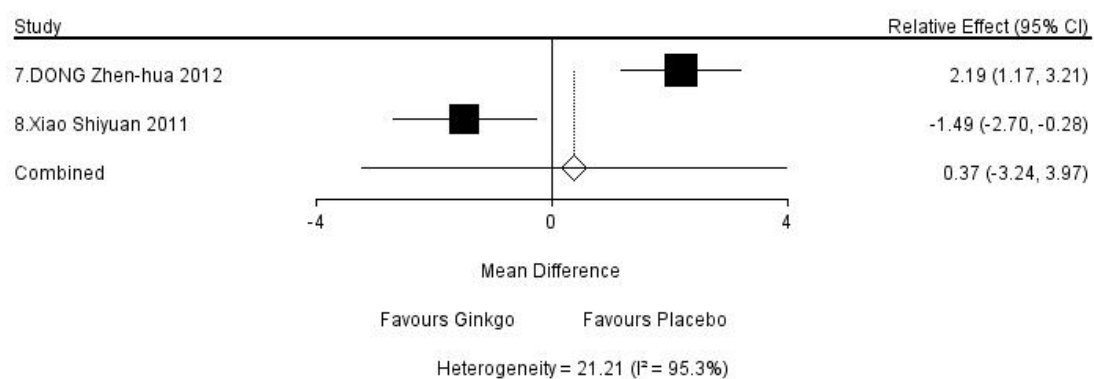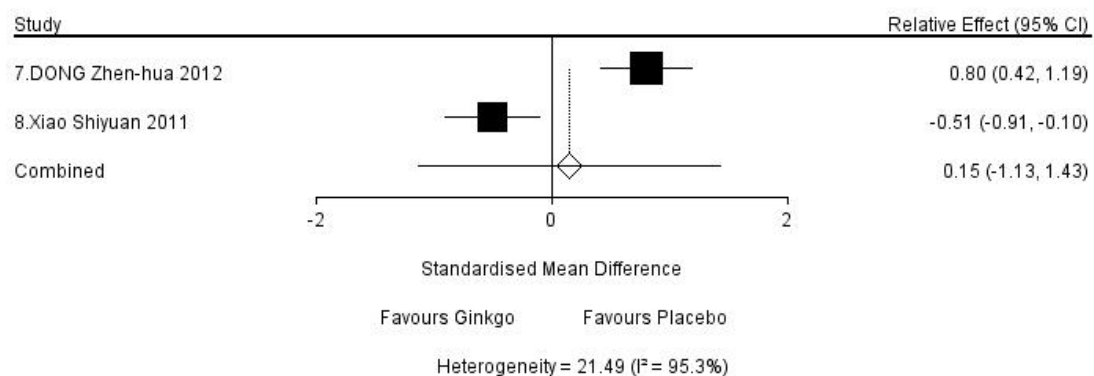

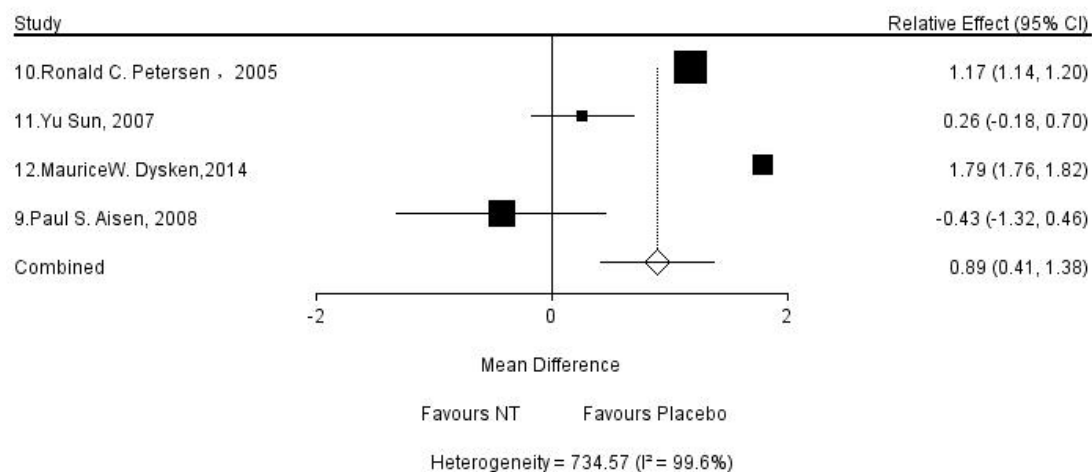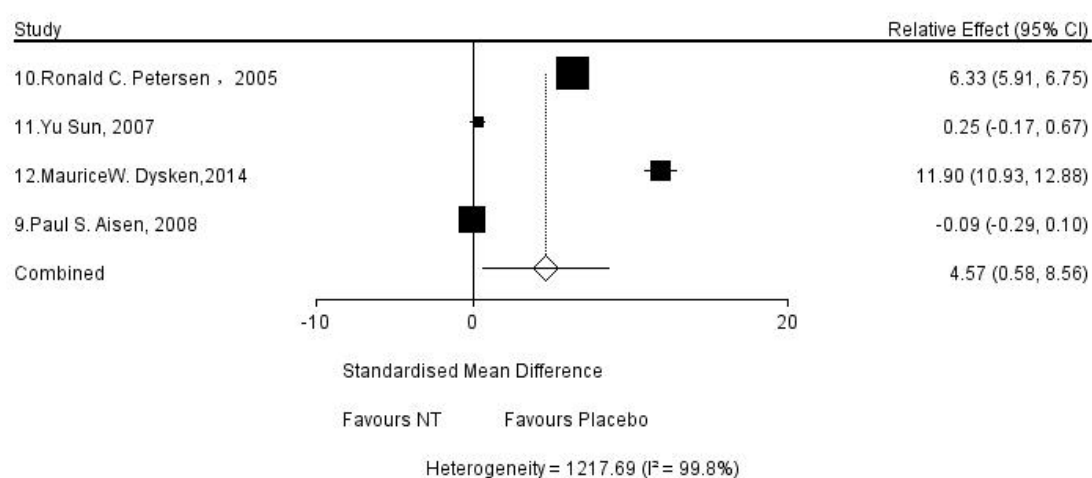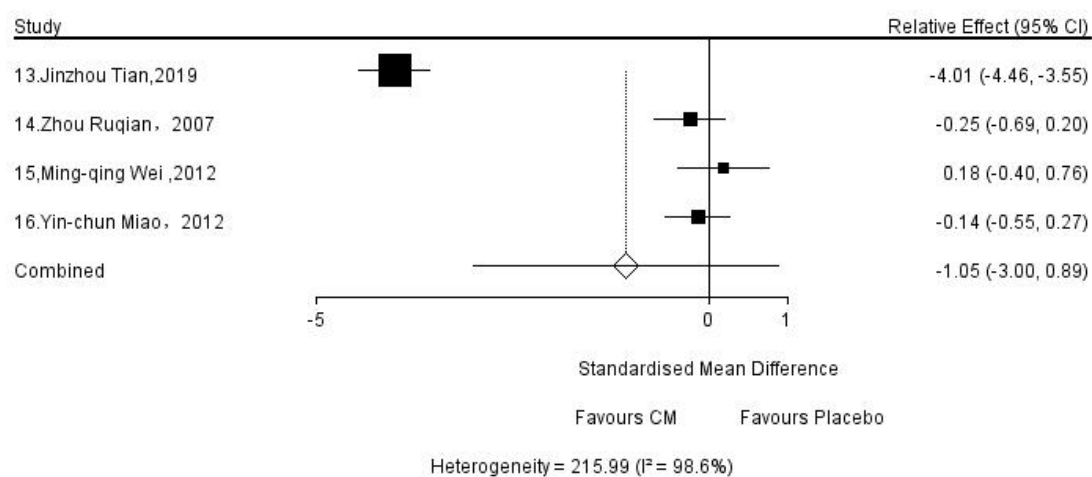

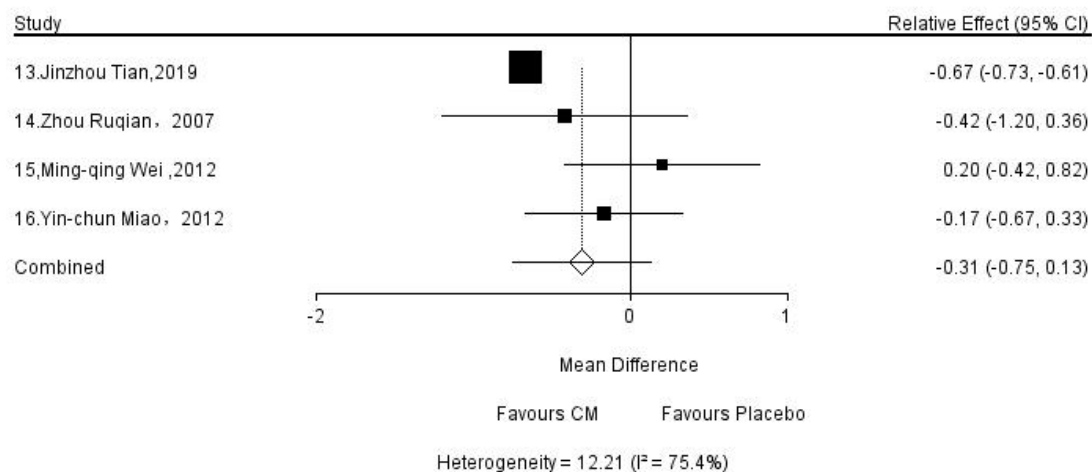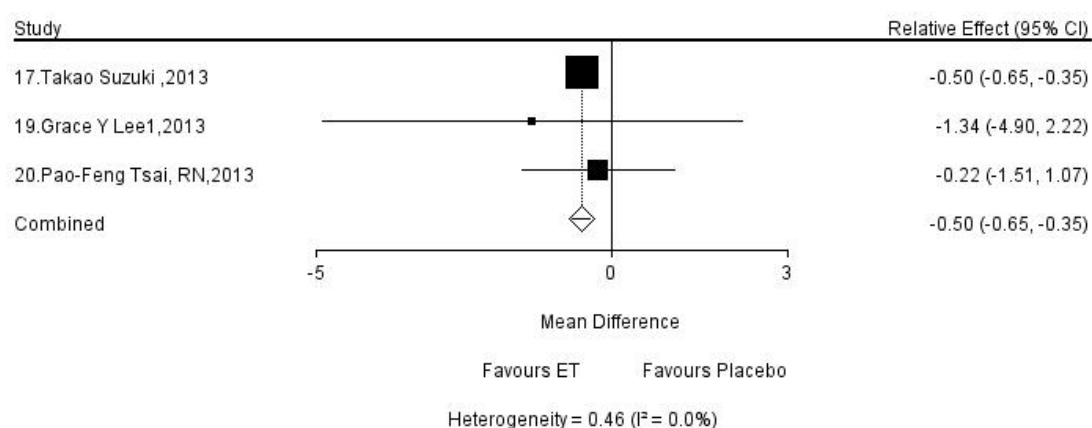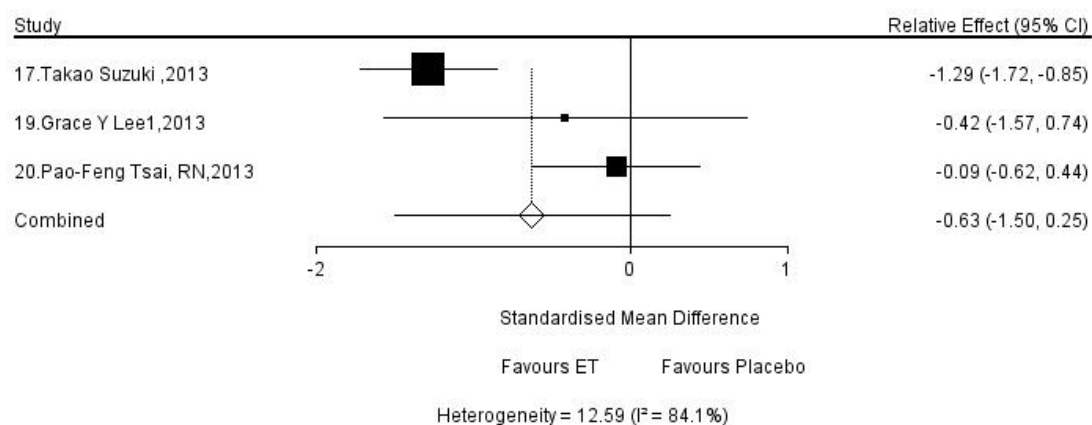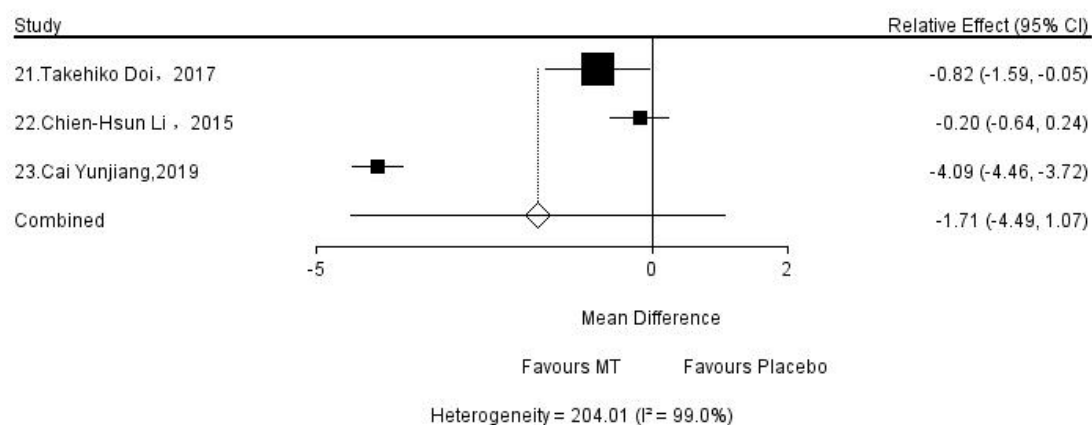

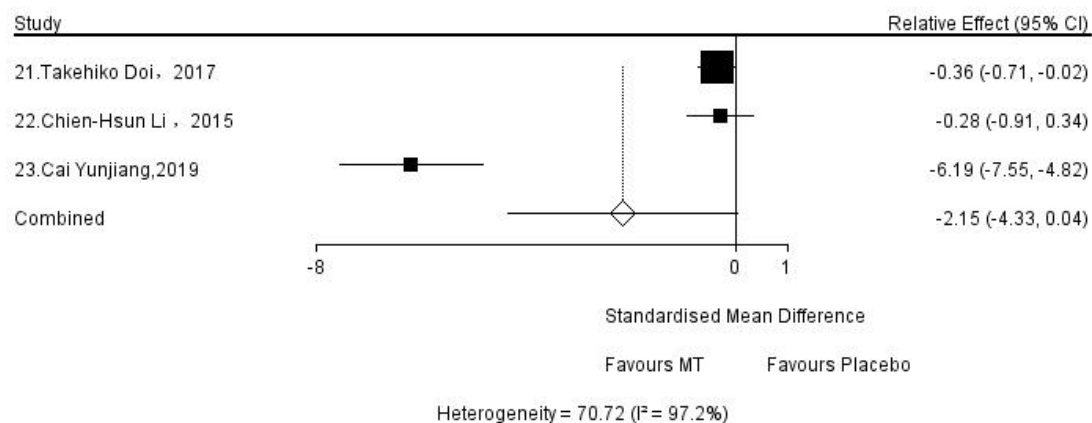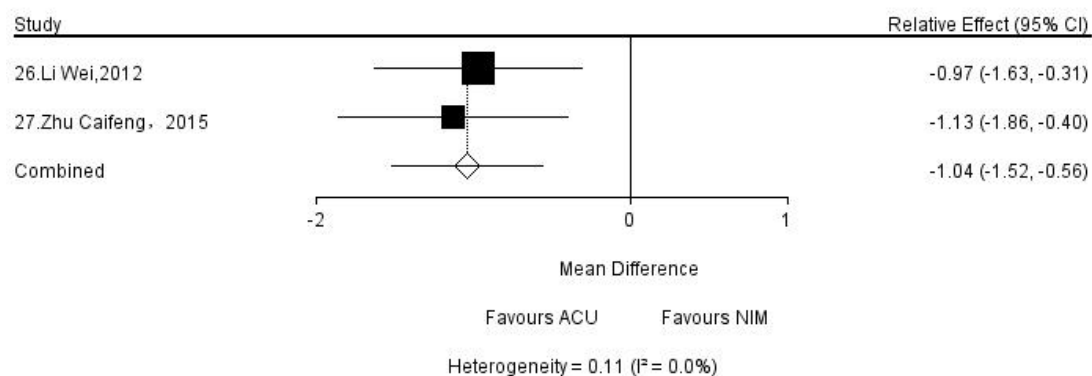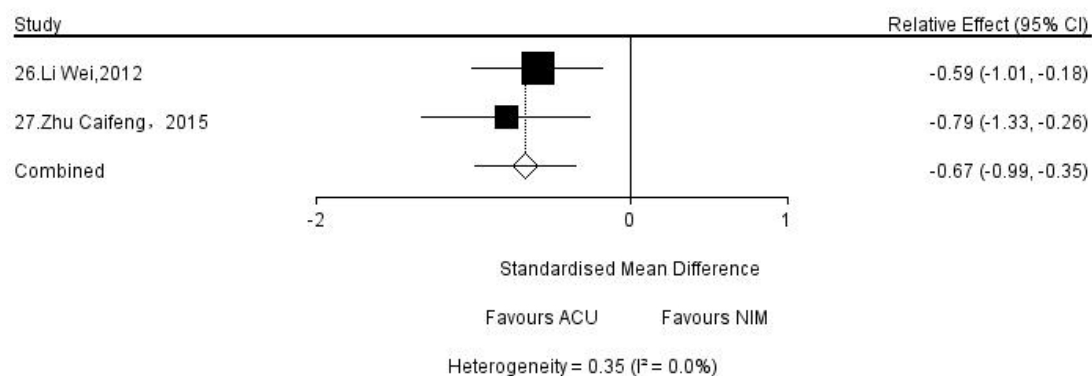

## Pair-wise analysis of ADAS-cog

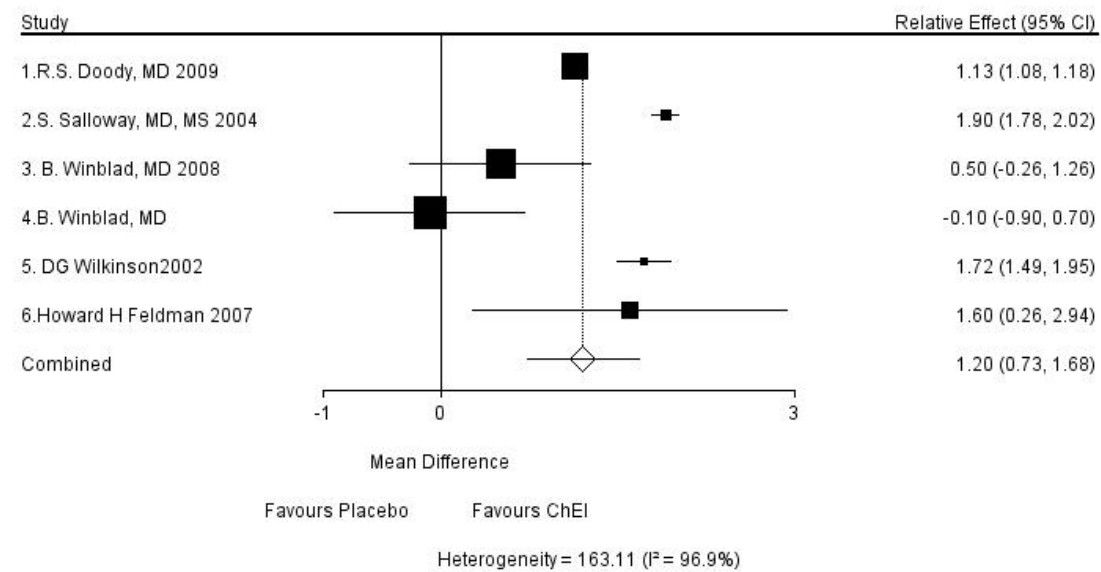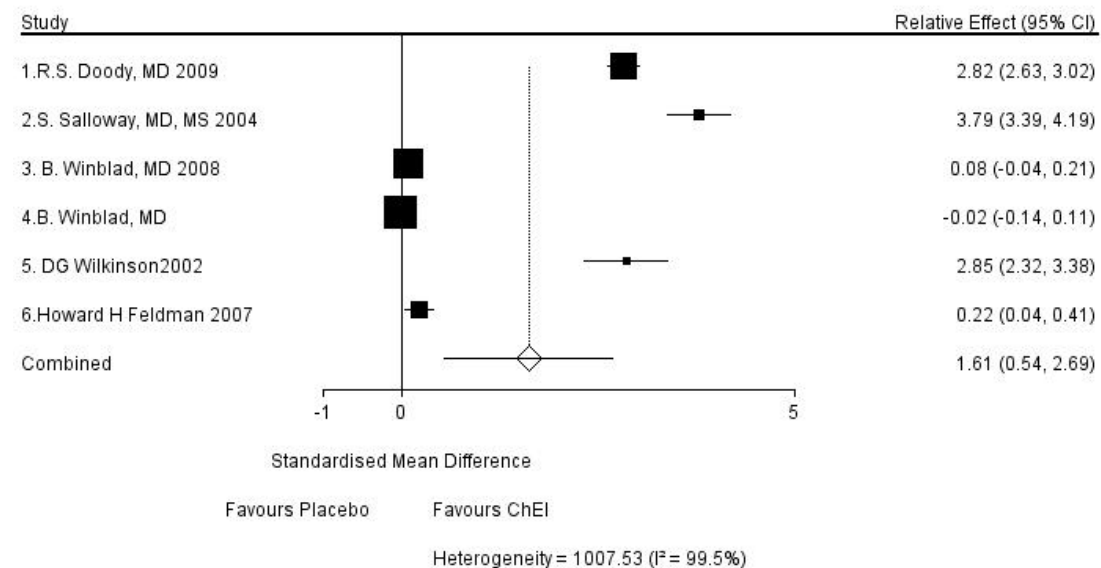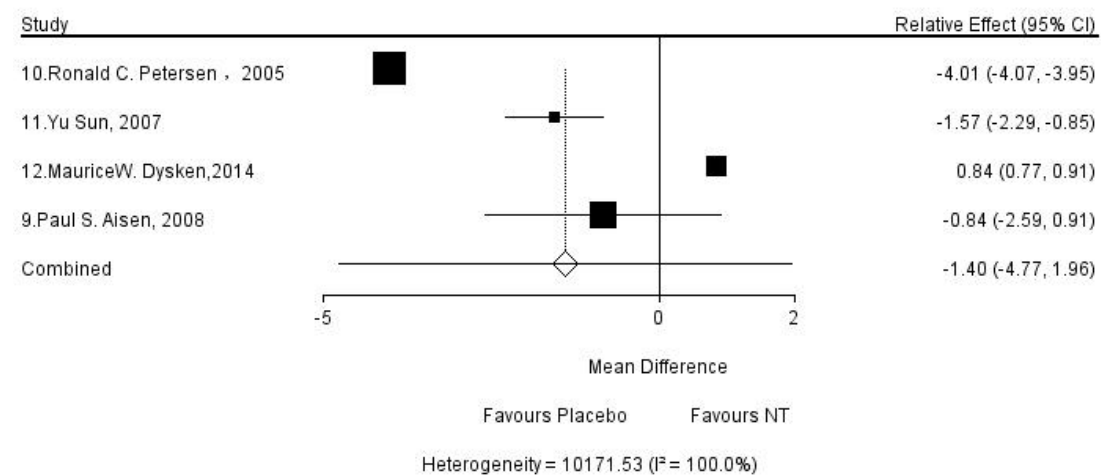

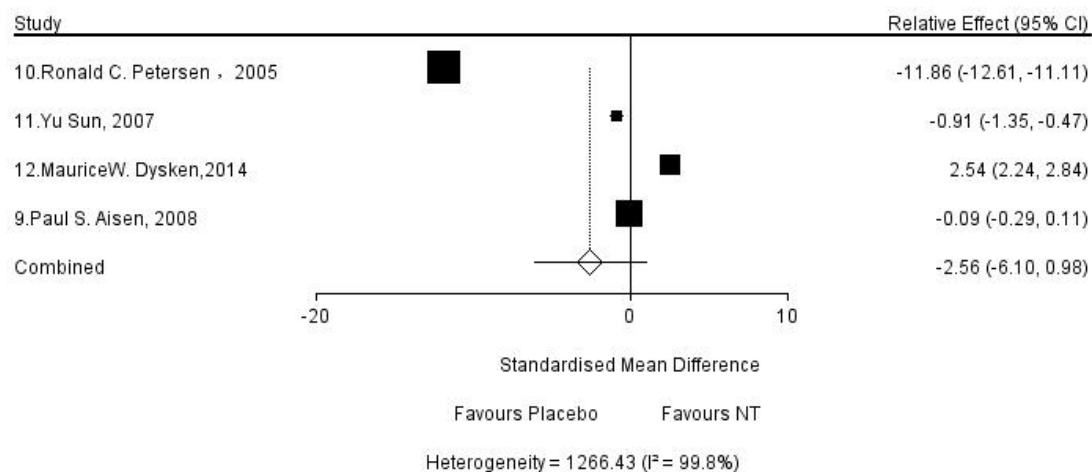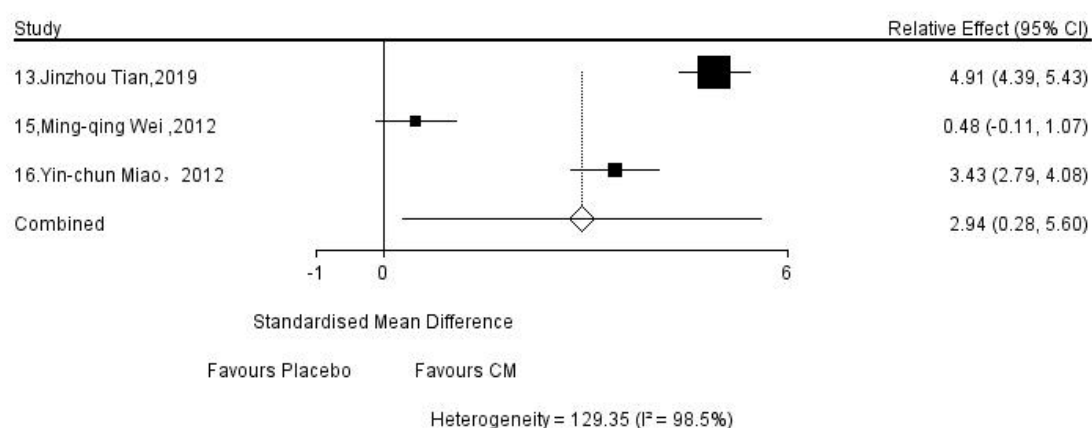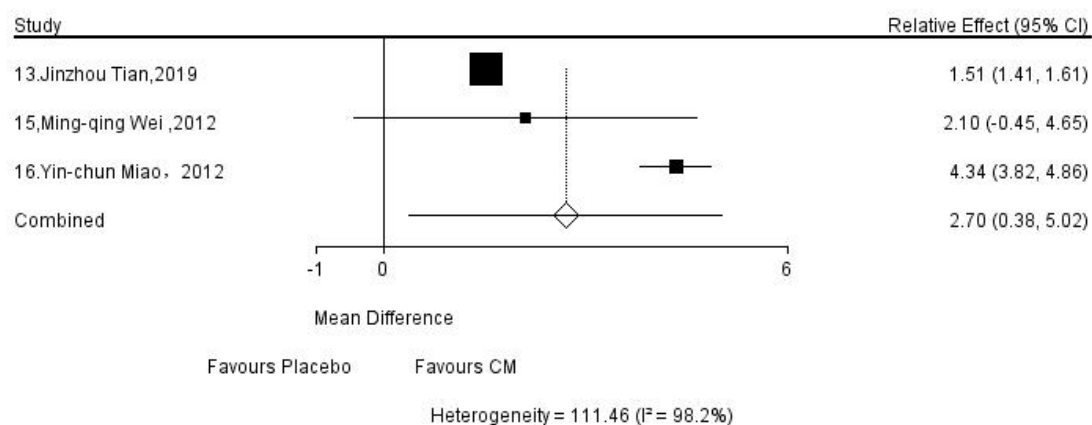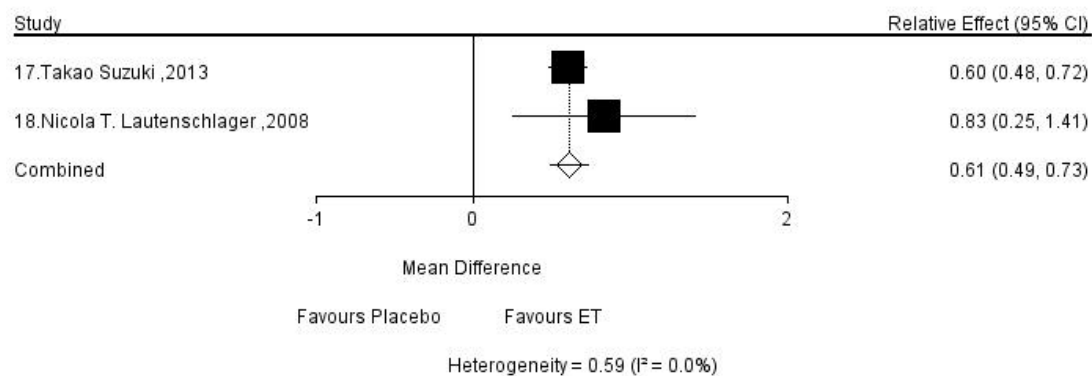

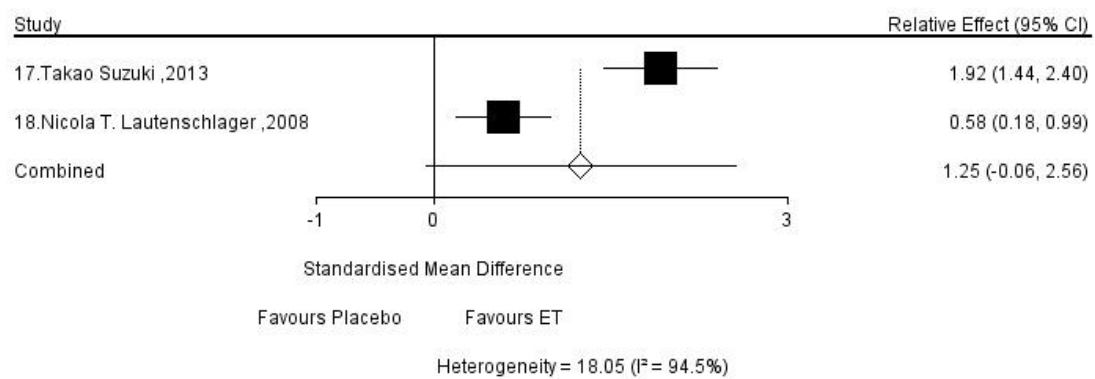

Supplement: Supplementary file 2 [file Data_Sheet_2.PDF]
